# Construction of Recombinant Marek’s Disease Virus Co-Expressing VP1 and VP2 of Chicken Infectious Anemia Virus

**DOI:** 10.3390/vaccines12091047

**Published:** 2024-09-13

**Authors:** Kai Li, Yongzhen Liu, Changjun Liu, Yanping Zhang, Hongyu Cui, Xiaole Qi, Jiayong Zhang, Jia Xu, Suyan Wang, Yuntong Chen, Yulu Duan, Yulong Gao, Xiaomei Wang

**Affiliations:** 1 Avian Immunosuppressive Diseases Division, State Key Laboratory for Animal Disease Control and Prevention, Harbin Veterinary Research Institute, Chinese Academy of Agricultural Sciences, Harbin 150001, China; likai01@caas.cn (K.L.); liuyongzhen@caas.cn (Y.L.); liuchangjun@caas.cn (C.L.); zhangyanping@caas.cn (Y.Z.); cuihongyu@caas.cn (H.C.); qixiaole@caas.cn (X.Q.); wangsuyan@caas.cn (S.W.); chenyuntong@caas.cn (Y.C.); duanyulu@caas.cn (Y.D.); 2 Institute of Urban Agriculture, Chinese Academy of Agricultural Sciences, Chengdu 610299, China; 3 Chengdu National Agricultural Science and Technology Center, Chengdu 610299, China; 4 Heilongjiang Provincial Center for Animal Disease Prevention and Control, Harbin 150049, China; cadc0451@126.com (J.Z.); saraxu1980@126.com (J.X.); 5 Jiangsu Co-Innovation Center for Prevention and Control of Important Animal Infectious Disease and Zoonoses, Yangzhou University, Yangzhou 225001, China

**Keywords:** Marek’s disease virus, chicken infectious anemia virus, VP1, VP2, vaccine

## Abstract

The chicken infectious anemia virus (CIAV) has been reported in major poultry-producing countries and poses a significant threat to the poultry industry worldwide. In this study, two Marek’s disease virus (MDV) recombinants, rMDV-CIAV-1 and rMDV-CIAV-2, were generated by inserting the CIAV VP1 and VP2 genes into the MDV vaccine strain 814 at the US2 site using the fosmid-based rescue system. For rMDV-CIAV-1, an internal ribosome entry site was inserted between VP1 and VP2, so that both proteins were produced from a single open reading frame. In rMDV-CIAV-2, VP1 and VP2 were cloned into different open reading frames and inserted into the MDV genome. The recombinant viruses simultaneously expressed VP1 and VP2 in infected chicken embryo fibroblasts and exhibited growth kinetics similar to those of the parent MDV. The two recombinant viruses induced antibodies against CIAV in chickens. A single dose of the recombinant viruses provided strong protection against CIAV-induced anemia in chickens. These recombinant VP1- and VP2-expressing MDVs are potential vaccines against CIAV in chickens.

## 1. Introduction

The chicken infectious anemia virus (CIAV) is an immunosuppressive virus in chickens that causes chicken infectious anemia (CIA), a disease characterized by aplastic anemia and systemic lymphoid tissue atrophy [1]. CIAV is a single-stranded, circular, negative-stranded DNA virus with a size of approximately 2.3 kb, and three viral proteins, VP1, VP2, and VP3, are encoded by three partially overlapping open reading frames (ORFs). VP1 functions as a structural protein and is the main immunogen, which triggers chickens to produce neutralizing antibodies [2]. VP2 is an essential scaffold protein involved in the assembly of CIAV, helping VP1 form the correct conformation. VP1 and VP2 work together to induce immune responses in the host [3]. VP3 is a non-structural protein that can induce apoptosis of infected chicken cells [4,5].

CIAV was first reported in Japan in 1970 and has since become widespread worldwide [6,7,8]. CIAV can persistently spread in chicken flocks through vertical or horizontal transmission and is difficult to eradicate [9]. This disease mainly affects chicks aged 1 to 3 weeks [9,10], while adult chickens typically experience subclinical infections. Additionally, CIAV can directly reduce the protective efficacy of certain vaccines and enhance the invasion of other viral and bacterial pathogens, resulting in growth and developmental disorders in infected chickens [11]. Currently, except in China, CIAV commercial attenuated vaccines are widely used in breeding flocks. However, these attenuated live vaccines have moderate virulence and may damage the immune organs of chickens [12,13]. Although CIAV inactivated vaccines can provide moderate protection in breeding chickens [14], they have not yet been applied for large-scale commercial use due to the inability of the virus to grow to high titers in vitro, which is required for the production of inactivated vaccines. Therefore, developing safer and more effective vaccines is crucial for the prevention of CIAV infection.

Marek’s disease (MD) is a highly contagious malignant T-cell lymphoma caused by the Marek’s disease virus (MDV), which primarily affects chicken flocks [15]. MDV has a large genome that contains multiple regions that are non-essential for viral replication and thus suitable for the insertion of foreign genes, making it an effective vector for recombinant vaccines [16,17]. In previous studies, several MDV-vectored vaccines against different poultry diseases had been developed. The recombinant MDV expressing the VP2 protein of the infectious bursal disease virus (IBDV) conferred full protection against very virulent IBDV and MDV in chickens [18]. With the MDV serotype 1 vaccine CVI988 as a vector, the recombinant virus expressing the F gene was constructed and efficiently protected commercial chickens from the Newcastle disease virus (NDV) challenge [19]. Also, the MDV Md5 strain with both the Meq oncogene deleted has been used to develop recombinant vaccines expressing glycoproteins B and J of the infectious laryngotracheitis virus [20].

The mixed infection of CIAV and MDV is becoming increasingly common in poultry, leading to increasing economic losses. Developing a bivalent vaccine that can simultaneously protect against CIAV and MDV is the most economical way to reduce losses in the chicken industry due to these diseases. However, there are no commercially used vaccines against both MDV and CIAV infections in chickens. Therefore, in the present study, we inserted the VP1 and VP2 genes of CIAV into the genome of the MDV vaccine strain through overlapping fosmid DNA transfection and constructed two recombinant MDVs. Further evaluation of the recombinant virus was conducted in vitro and in vivo to detect its antigen expression, replication ability, genetic stability, and protective efficacy against CAIV in chickens. These rMDVs were shown to be efficient for the prevention of CIAV infection and have significant potential for use as MDV-vectored CIAV vaccines to combat CIA in chickens.

## 2. Materials and Methods

### 2.1. Animals and Ethics Statement

The specific pathogen-free (SPF) chickens and fertilized SPF eggs used in this study were purchased from the State Resource Center of the Animal Laboratory for Poultry (Harbin, China). The chicken embryo fibroblasts (CEFs) were prepared using 10-day-old SPF chicken embryos. This study was conducted strictly in accordance with the recommendations of the Guidelines for the Care and Use of Laboratory Animals, issued by the Ministry of Science and Technology of China. The use of SPF embryos, chickens, and animal experiments conducted were approved by the Animal Ethics Committee of the Harbin Veterinary Research Institute of the Chinese Academy of Agricultural Sciences and conducted in accordance with the animal ethics guidelines and approved protocols (SYXK (Hei) 2017-009).

### 2.2. Viruses and Cells

The MDV serotype 1 (MDV1) 814 vaccine strain [21] was used as the parent virus for producing the recombinant MDVs. The CIAV DA strain was used as a challenge virus. The rescued MDVs were propagated in CEFs prepared from 10-day-old SPF chicken embryos.

### 2.3. Construction of Fosmids with Insertion of CIAV VP1 and VP2 Genes

**Figure 1:**
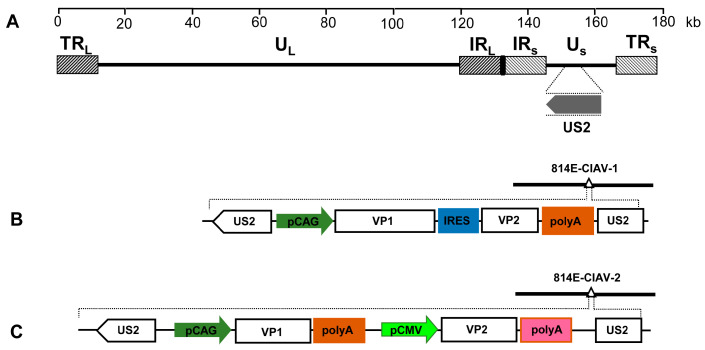
Construction of fosmids containing CIAV VP1 and VP2 genes. (**A**) The genomic structure of the MDV vaccine strain 814. (**B**) A schematic diagram of the recombinant fosmid 814E-CIAV-1 with the VP1-IRES-VP2 cassette inserted within the US2 gene in the MDV genome. (**C**) A schematic diagram of the recombinant fosmid 814E-CIAV-2 with the VP1 and VP2 expression cassette inserted within the US2 gene in the MDV genome.

In the preliminary study [22], five fosmids (814A, 814B, 814C, 814D, and 814E) containing sequences spanning the entire genome of MDV vaccine strain 814 (GenBank accession number JF742597) were constructed for the insertion of the CIAV VP1 and VP2 genes (Figure 1A). To promote the insertion of foreign genes into the MDV genome, a dual-selection marker cassette containing a kanamycin resistance gene (KanR) and the ccdB gene flanked by attR1 and attR2 sequences were inserted into the recombinant fosmid 814E at the US2 site through Red/ET recombination in accordance with the instructions of the counter-selection BAC modification kit (Gene Bridges GmbH, Heidelberg, Germany). The modified fosmids with selection markers were designated 814E-Kan/ccdB.

The gene fragment VP1-IRES-VP2, containing the CIAV VP1 and VP2 genes and the internal ribosome entry site (IRES), was cloned into a pCAGGS vector with the CAG promoter. The constructed VP1-IRES-VP2 cassette was then used to replace gus in the pENTR-gus plasmid (Invitrogen, Carlsbad, CA, USA), and the resultant entry plasmid was designated pENTR-VP12-1. Then, the plasmid pENTR-VP12-1 was generated by replacing gus in pENTR-gus (Invitrogen) with the constructed VP1-IRES-VP2 cassette. To insert the VP1-IRES-VP2 cassette into the MDV genome, the modified fosmid 814E-Kan/ccdB was mixed with the entry plasmid pENTR-VP12-1, followed by treatment with the LR Clonase II (Invitrogen). The mixtures were transferred into the *Escherichia coli* EPI300-T1 competent cells. The resulting fosmids with the VP1-IRES-VP2 cassette insertion were designated 814E-CIAV-1 (Figure 1B). To construct the recombinant fosmid 814E-CIAV-2, the VP1 gene under the control of the CAG promoter and the VP2 gene under the control of the CMV promoter were simultaneously cloned into pENTR-gus and then inserted into the US2 gene in fosmid 814E (Figure 1C).

### 2.4. Rescue of Recombinant Viruses from Overlapping Fosmid DNAs

A set of five fosmids with or without VP1 and VP2 insertions, covering the entire genome of the MDV 814 strain, was used to rescue the recombinant viruses. The virus DNA insertion fragments were released from purified fosmids by NotI digestion and transfected into CEFs using the calcium phosphate method. Four days after transfection, the cells were trypsinized and seeded into a 100-mm culture dish, and the cytopathic effect (CPE) was monitored. CPE-positive samples were collected and characterized with electron microscopy. To verify the correct insertion of VP1 and VP2 genes in the desired sites of the MDV genome, genomic DNA was isolated from the rescued viruses and analyzed by PCR and sequencing.

### 2.5. Confirmation of VP1 and VP2 Gene Expression

The expression of CIAV VP1 and VP2 by recombinant MDVs was confirmed by indirect immunofluorescence detection. In short, CEFs were infected with the rescued viruses in six-well tissue culture plates and cultured for 4 days. Then the culture medium was removed, and the cells were fixed with absolute ethanol at room temperature for 20 min. The fixed cells were incubated with mouse anti-VP1 monoclonal antibody (MAb) (1:1000) or mouse anti-VP2 MAb (1:1000) at 37 °C for 60 min, followed by a reaction with the FITC-conjugated goat anti-mouse IgG antibody (Sigma, St. Louis, MO, USA) (1:200) at 37 °C for 60 min. After five washes, the cells were observed using a fluorescence microscope.

### 2.6. Growth and Stability of Recombinant Viruses

The replication characteristics of the recombinant MDVs were investigated in CEFs. Briefly, cells cultured in six-well plates were inoculated with 100 PFU of the rescued viruses. The infected cells were collected every 24 h, and the fresh CEFs were inoculated with the serial dilutions of the harvested cells. The plaques at different dilutions were counted after 5 days of culturing. To evaluate the genetic stability of recombinant MDVs, the viruses were passaged 20 times in CEFs. The inserted genes were detected by PCR and sequencing using genomic DNA purified from 10^5^ PFU of each virus. Immunofluorescence analysis using the monoclonal antibodies against VP1 and VP2 was conducted to confirm the expression of VP1 and VP2 in the infected cells.

### 2.7. Serological Tests and Protection against the CIAV Challenge

To evaluate the protective efficacy of the recombinant viruses against a challenge with the virulent CIAV, each group of 10 SPF chickens was inoculated subcutaneously on the back of the neck with 2000 PFU of the rescued viruses at 1 day of age and intramuscularly challenged with 1000 EID_50_ CIAV DA strain at 21 days of age. Ten unvaccinated chickens were challenged in parallel as the challenge control, and ten unvaccinated and unchallenged chickens were used as mock controls. A total of 14 days after the viral challenge infection, a clinical observation was conducted on the challenged chickens, and the mortality rate was recorded. The anticoagulated blood of each group was collected 14 days post-viral challenge, and the hematocrit (HCT) was calculated. In addition, the thymus from each group of chickens was collected and weighed, and the thymus index was calculated using the following formula (TBIX): (thymus: body weight ratio)/(thymus: body weight ratio of a healthy group). Thymus with a TBIX score below 0.70 is considered atrophied. Serum samples were collected from chickens 3 weeks after vaccination and 2 weeks post-challenge, and ELISA testing was performed using a commercial ELISA kit (IDEXX, Westbrook, ME, USA).

### 2.8. Statistical Analysis

The results are presented as the mean ± standard deviation (SD). A one-way analysis of variance (ANOVA) and t-tests were used to evaluate the statistical differences among groups using SPSS (version 17.0; SPSS Inc., Chicago, IL, USA). The statistical significance was set at *p* < 0.05 for all tests.

## 3. Results

### 3.1. Generation of Recombinant MDVs Expressing CIAV VP1 and VP2 Genes

**Figure 2:**
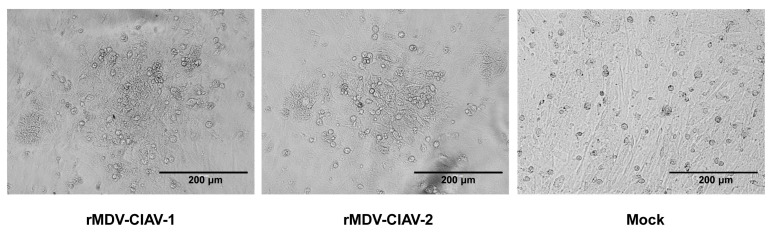
The cytopathic effect (CPE) induced by the recombinant Marek’s disease virus (MDV) containing the VP1 and VP2 genes in chicken embryo fibroblasts (CEFs). The recombinant MDVs were inoculated in CEFs for 4–5 days before the CPE was checked. Bar length, 200 μm.

The VP1-IRES-VP2 expression cassette was inserted into the US2 gene of the MDV genome, and the resultant recombinant fosmid 814E-CIAV-1 was co-transfected with the other parental fosmids into CEFs to construct rMDV-CIAV-1. For rMDV-CIAV-2, the VP1 and VP2 genes were cloned under the control of CAG and CMV promoters, respectively, and the VP1 and VP2 cassettes were simultaneously inserted into the MDV genome. After one blind passage in CEFs, typical plaques of MDV appeared in CEFs transfected with the DNA combination (Figure 2). The insertion of the VP1 and VP2 genes at the correct site was confirmed using PCR and sequencing.

**Figure 3:**
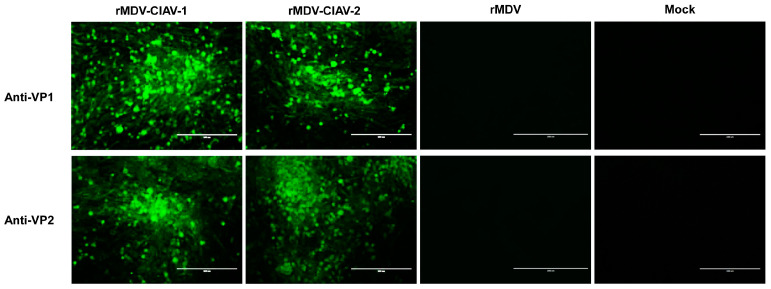
Detection of VP1 and VP2 expression by the recombinant viruses. The rescued viruses (rMDV-CIAV-1, rMDV-CIAV-2) or the parental virus (rMDV) chicken embryo fibroblasts (CEFs) in six-well tissue culture plates were infected for 4 days, and the expression of VP1 and VP2 was detected in an indirect immunofluorescence assay with anti-VP1 and anti-VP2 antibodies (1:1000). Bar length, 200 μm.

The co-expression of the VP1 and VP2 proteins by recombinant viruses in the infected cells was confirmed using an indirect immunofluorescence assay with anti-VP1 and anti-VP2 antibodies. The results showed that cells infected with rMDV-CIAV-1 or rMDV-CIAV-2 reacted with both anti-VP1 and anti-VP2 antibodies, emitting a green fluorescent signal (Figure 3). In contrast, the cells inoculated with the parental virus did not react with these antibodies. These results indicate that recombinant MDVs co-expressing the VP1 and VP2 genes of CIAV were successfully generated.

### 3.2. Growth Kinetics and Genetic Stability of Recombinant Marek’s Disease Viruses

**Figure 4:**
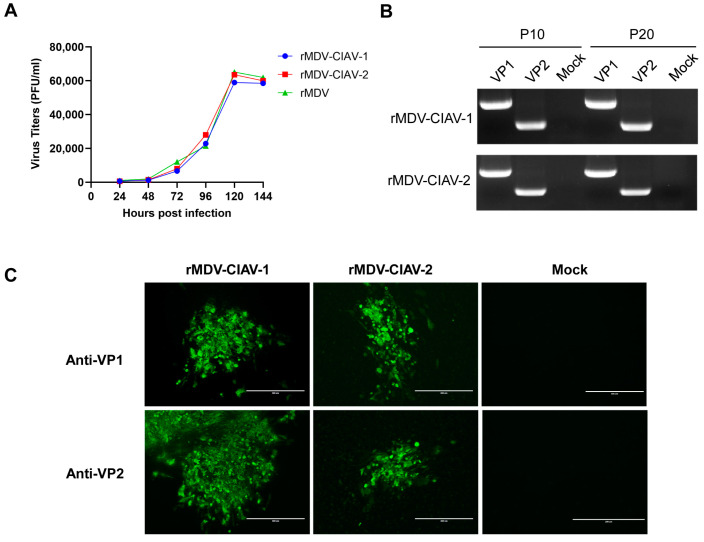
Growth kinetics and genetic stability of the recombinant Marek’s disease viruses (MDVs) in chicken embryo fibroblasts (CEFs). (**A**) Comparison of the replication kinetics of the recombinant MDVs and the parental virus (rMDV) in CEFs. (**B**) PCR detection of the VP1 and VP2 genes inserted in the recombinant MDVs passaged 10 (P10) and 20 (P20) times in CEFs. (**C**) Confirmation of VP1 and VP2 expression by the recombinant MDVs passaged 20 times in CEFs with immunofluorescence assay. Bar length, 400 μm.

The replication of recombinant viruses was analyzed and compared with that of the parental viruses in CEFs. CEF cultures infected with the viruses were collected at different time points for titer determination. As shown in Figure 4A, the replication kinetics and magnitude of the two recombinant viruses, rMDV-CIAV-1 and rMDV-CIAV-2, were quite similar to their parental virus MDV1 814 vaccine strain (rMDV), indicating that inserting VP1 and VP2 genes at the US2 site did not significantly affect the replication of the MDV vaccine strain in CEFs.

To investigate whether the inserted VP1 and VP2 genes can be stably maintained in the recombinant viruses, these viruses were passaged 20 times in CEFs. PCR detection revealed that the VP1 and VP2 genes in the two recombinant viruses were stably maintained during passaging (Figure 4B), and the sequences were correct. After 20 passages, immunofluorescence confirmed that VP1 and VP2 proteins were stably expressed in cells infected with the recombinant virus (Figure 4C).

### 3.3. Antibody Responses against Chicken Infectious Anemia Virus Induced by Recombinant Marek’s Disease Virus Vaccines in Chickens

**Figure 5:**
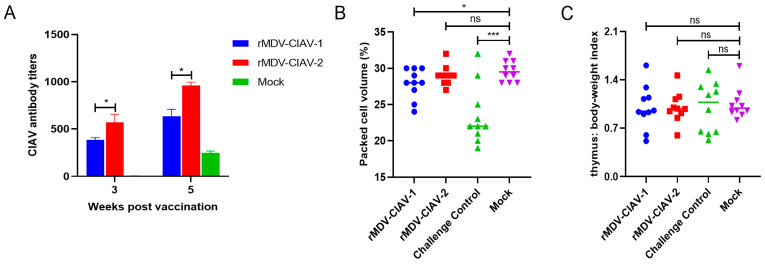
Antibody responses and protective efficacy induced by the recombinant Marek’s disease viruses (MDVs) in chickens. Groups of 10 chickens were inoculated with 2000 PFU of the recombinant MDVs and challenged with the chicken infectious anemia virus (CIAV) at 21 days post-vaccination. (**A**) The sera samples were collected 3 weeks post-vaccination and 2 weeks post-challenge to detect antibodies against CIAV using ELISA. (**B**) Hematocrit (HCT) of chickens in each group after 2 weeks post-challenge with CIAV. (**C**) The thymus index of chickens in each group after 2 weeks post-challenge with CIAV. *, *p* < 0.05; ***, *p* < 0.001; ns, no significant difference.

To evaluate the antibody responses induced by recombinant MDVs against CIAV, groups of ten chickens were inoculated with 2000 PFU of the rescued viruses. Antibody responses against CIAV in chickens vaccinated with the recombinant viruses were detected using ELISA. As shown in Figure 5A, both recombinant viruses induced antibody responses against CIAV in chickens. The antibody level against CIAV induced by rMDV-CIAV-2 was significantly higher than that against rMDV-CIAV-1 at 3 weeks post-vaccination (*p* < 0.05). Two weeks post-challenge, the antibodies against CIAV significantly increased, and the antibody titers in the rMDV-CIAV-2 group were higher than those in the rMDV-CIAV-1 group (*p* < 0.05).

### 3.4. Protective Efficacy against Chicken Infectious Anemia Virus Challenge in Chickens

To evaluate the protective efficacy of the recombinant viruses, chickens were challenged with virulent CIAV 21 days post-vaccination, and the HCT and thymus index of the chickens were determined. Chickens in the rMDV-CIAV-1 and rMDV-CIAV-2 vaccination groups showed no clinical signs of disease during the 2-week observation period. As shown in Figure 5B, the HCT value of the chickens in the challenge control group was significantly reduced, with eight out of ten chickens showing an HCT value lower than 27%. In the rMDV-CIAV-1 group, two out of ten chickens had an HCT value lower than 27%, and no chickens in the rMDV-CIAV-2 group showed an HCT value lower than 27%. These results indicated that rMDV-CIAV-2 provides protection against CIAV-induced anemia. After challenge with CIAV, four chickens in the challenged control group showed thymus atrophy with a TBIX < 0.7, and eight and nine chickens in the rMDV-CIAV-1 and rMDV-CAIV-2 groups, respectively, were protected against thymus atrophy caused by CIAV infection (Figure 5C).

## 4. Discussion

Recently, CIAV infections have spread globally, causing serious economic losses. A study reported that the positive detection rate of CIAV in 404 serum samples from 11 poultry farms in India was 86.88% [23]. In northern Vietnam, 62.2% of positive tests from 64 farms have been reported [6]. Epidemiological investigations have shown that the infection rate of CIAV in chicken flocks in China ranges from 40 to 70%, particularly in young chicks aged 1–3 weeks [24]. From 2017 to 2020, six types of avian immunosuppressive pathogens were detected in 1187 clinical samples from major poultry farms across the country, with CIAV having the highest detection rate [25]. Owing to the prevalence of CIAV infection in chickens, the development of a safe and effective vaccine against CIAV is urgently needed.

Several traditional vaccines have been reported to be effective against CIAV infection. Live attenuated vaccines trigger a strong immune response and protect against CIAV; however, such vaccines have the risk of reverting to their virulent nature and being horizontally transmitted to chickens [26]. Inactivated vaccines have been regarded as safe, though they induce a low immune response. Additionally, since CIAV has a low replication titer in chicken embryos and MDCC-MSB1 cells, the inactivated vaccines against CIAV have not been applied to large-scale commercial use because of their high production costs. Therefore, future vaccine designs should consider improving the safety and induction of neutralizing antibodies against CIAV.

As the major structural capsid protein of CIAV, VP1 plays an important role in antigenicity and induces neutralizing antibodies in the host [27]. Non-structural VP2 acts as a scaffold protein to assist in the correct assembly of VP1 [28,29]. Several genetically engineered vaccines are efficient against CIAV by co-expressing CIAV VP1 and VP2. A CIAV DNA vaccine encoding the VP1 and VP2 genes induced moderately protective ELISA antibody titers when administered to SPF chicks [26]. Tseng et al. (2019) [30] developed a subunit vaccine exploiting the CAV structural proteins VP1 and VP2 and confirmed the ability of recombinant VP1 to generate self-assembling virus-like particles. Newcastle disease virus (NDV) was previously used as a viral vector to deliver the VP1 and VP2 genes of CIAV to generate a bivalent vaccine candidate against these diseases in chickens [31]. The bivalent vaccine candidate elicited robust humoral and cell-mediated immune responses when administered to SPF chickens at primary and booster doses.

MDV is a useful vector for the construction of recombinant vaccines [16,17]. Since MDV is transmitted from cell to cell, it is not susceptible to maternal antibodies. The persistent infection of MDV makes MDV-vectored vaccines have a long immune duration. In the present study, the VP1 and VP2 genes of CIAV were co-inserted into the MDV genome, either within a single cassette containing an IRES element or cloned into two expression cassettes under the CAG and CMV promoters, respectively. The two recombinant MDVs were capable of co-expressing the VP1 and VP2 genes, which is important for the correct formation of VP1 and induction of neutralizing antibodies. The recombinant MDVs showed replication levels in CEFs compared to those of the parental virus, indicating that the insertion of VP1 and VP2 into the MDV genome did not significantly affect viral replication. The rMDV-CIAV-2 virus with VP1 and VP2 co-expressed in two different cassettes induced higher antibody titers against CIAV than rMDV-CIAV-1. After being challenged with the virulent CIAV, the rMDV-CIAV-2 vaccine virus provided full protection against CIAV-induced anemia. Since the age-dependent pathogenicity of CIAV, no dead chickens were observed after the CIAV challenge; however, four out of ten chickens in the challenge control group showed thymus atrophy, and only one chicken in the rMDV-CIAV-2 group showed thymus atrophy. Further studies are needed to analyze the duration of immunity of the recombinant viruses and optimize the vaccination doses of the recombinant viruses to achieve better protection against CIAV infection. Also, the protection efficacy of the recombinant viruses against MDV infection needs to be assessed further.

## 5. Conclusions

In summary, we generated recombinant MDVs expressing CIAV VP1 and VP2 genes. These rMDVs demonstrated good immunogenicity against CIAV in chickens. These recombinant viruses have significant potential for use as MDV-vectored CIAV vaccines to combat CIAV infection in chickens.

## Data Availability

Data can be requested by writing to the author.
